# MiR-199b-5p Suppresses Tumor Angiogenesis Mediated by Vascular Endothelial Cells in Breast Cancer by Targeting ALK1

**DOI:** 10.3389/fgene.2019.01397

**Published:** 2020-01-30

**Authors:** Xiao Lin, Wuxia Qiu, Yunyun Xiao, Jianhua Ma, Fang Xu, Kewen Zhang, Yongguang Gao, Qiang Chen, Yu Li, Hui Li, Airong Qian

**Affiliations:** ^1^ Laboratory for Bone Metabolism, Key Laboratory for Space Biosciences and Biotechnology, School of Life Sciences, Northwestern Polytechnical University, Xi’an, China; ^2^ Research Center for Special Medicine and Health Systems Engineering, School of Life Sciences, Northwestern Polytechnical University, Xi’an, China; ^3^ NPU-UAB Joint Laboratory for Bone Metabolism, School of Life Sciences, Northwestern Polytechnical University, Xi’an, China; ^4^ State Key Laboratory of Solidification Processing, Northwestern Polytechnical University, Xi’an, China; ^5^ Department of Joint Surgery, Honghui Hospital, Xi’an Jiaotong University, Xi’an, China

**Keywords:** angiogenesis, miR-199b-5p, ALK1, HUVECs, tumor growth, breast cancer

## Abstract

Angiogenesis is a crucial event during cancer progression that regulates tumor growth and metastasis. Activin receptor-like kinase 1 (ALK1), predominantly expressed in endothelial cells, plays a key role in the organization of neo-angiogenic vessels. Therapeutic targeting of ALK1 has been proposed as a promising strategy for cancer treatment, and microRNAs (miRNAs) are increasingly being explored as modulators of angiogenesis. However, the regulation of ALK1 by miRNAs is unclear. In this study, we identified that ALK1 is directly targeted by miR-199b-5p, which was able to inhibit angiogenesis *in vitro* and *in vivo*. Moreover, it was found that miR-199b-5p was repressed in breast cancer cells and its expression was decreased during the VEGF-induced angiogenesis process of human umbilical vein endothelial cells (HUVECs). Overexpression of miR-199b-5p inhibited the formation of capillary-like tubular structures and migration of HUVECs. Furthermore, overexpression of miR-199b-5p inhibited the mRNA and protein expression of ALK1 in HUVECs by directly binding to its 3’UTR. Additionally, overexpression of miR-199b-5p attenuated the induction of ALK1/Smad/Id1 pathway by BMP9 in HUVECs. Finally, overexpression of miR-199b-5p reduced tumor growth and angiogenesis in *in vivo*. Taken together, these findings demonstrate the anti-angiogenic role of miR-199b-5p, which directly targets ALK1, suggesting that miR-199b-5p might be a potential anti-angiogenic target for cancer therapy.

## Introduction

Angiogenesis is a physiological process that is defined as the formation of new blood vessels from pre-existing vessels (Ramjiawan et al., 2017). The process of angiogenesis is crucial not only for normal processes such as wound healing, tissue growth and regeneration, and placental development, but also for pathophysiological changes such as cancer progression (Petrovic, 2016). As early as 1971, Folkman postulated that angiogenesis is required for the growth of solid tumors (Folkman, 1971). Tumor tissues need oxygen and nutrients for growth and metastasis, which are provided by blood vessels (Rajabi and Mousa, 2017). In cancer progression, the imbalance between pro- and anti-angiogenic factors leads to abnormal homeostasis of blood vessels (Bergers and Benjamin, 2003). Several pro-angiogenic factors such as vascular endothelial growth factor (VEGF), vascular endothelial factor (TGF), and interleukin-8 (IL-8) increase the motility, proliferation, and survival of endothelial cells (He et al., 2014). Conversely, angiogenesis inhibitors such as angiostatin, endostatin, and interferons can promote tumor starvation and trigger cell death (Lin et al., 2016).

Transforming growth factor-β (TGF-β) is a multifunctional growth factor involved in the regulation of various cellular processes including proliferation, migration, differentiation, and synthesis of the extracellular matrix (Goumans et al., 2003; Finnson et al., 2008). TGF-β initiates cellular responses by binding to transmembrane kinases known as type I (activin receptor-like kinases ALK1–ALK7) and type II serine/threonine receptors (Ye et al., 2011). Among these signaling receptors, ALK1 (also known as ACVRL1) is predominantly expressed in endothelial cells (Roelen et al., 1997). Upon ligand binding, ALK1 is phosphorylated and forms a complex with TGF-β and its type II receptors, which subsequently phosphorylates the Smad proteins (Smad1/5/8), triggering transcriptional regulation of proangiogenic genes like Id1 (Goumans et al., 2002; Valdimarsdottir et al., 2002). Genetic studies have revealed that ALK1 plays a key role in vasculogenesis and organization of neo-angiogenic vessels (Hu-Lowe et al., 2011). Homozygous ALK1^−/−^ mice died at embryonic days 10–10.5 due to vascular and cardiac abnormalities (Oh et al., 2000). Additionally, ALK1 expression is upregulated in large arteries during tumor angiogenesis (Mitchell et al., 2010). Immunological sequestration of ALK1 ligands (using ALK1-Fc) or antibody against ALK1 inhibits tumor angiogenesis and tumor growth of mouse model of pancreatic cancer, breast cancer, and melanoma (Roman and Hinck, 2017). Therefore, ALK1 has emerged as a potential target for antiangiogenic therapy of tumors (Cunha and Pietras, 2011; Hawinkels et al., 2013; Cunha et al., 2015).

MicroRNAs (miRNAs) are small non-coding RNAs that regulate gene expression either by inhibiting the translation of targeted genes or by reducing the stability of their mRNA (Ambros, 2004). MiRNAs play essential and critical roles in biological processes such as cell-cycle regulation, stress responses, differentiation, migration, and tumor progression (Di Leva et al., 2012). Moreover, accumulating evidence indicates that miRNAs are important regulators of angiogenesis. For example, miR-524 suppresses angiogenesis and tumor growth by functionally inhibiting the translation of angiopoietin-2 (He et al., 2014). Similarly, miR-363 acts as a negative regulator of angiogenesis by attenuating the expression of the growth hormone receptor gene (Zhu et al., 2019). MiR-29b suppresses tumor growth accompanied by inhibition of angiogenesis and tumorigenesis by targeting Akt3 (Li et al., 2017). MiR-320 plays a critical role in suppressing tumor angiogenesis through the silencing of neuropilin 1 (Wu et al., 2014). However, the role of miRNAs in the regulation of angiogenesis is not well understood.

To explore the possibility that ALK1 expression is regulated by miRNAs, TargetScan, and miRanda were used to analyze the interactions between different miRNAs and the 3’UTR of ALK1. Several miRNAs, including miR-199b-5p, were found to have potential binding sites in the 3’UTR of ALK1. Many reports demonstrated that miR-199b-5p acts as a tumor suppressor (Lai et al., 2018; Wu et al., 2018), but its function in endothelial cells during angiogenesis is unclear. Therefore, we investigated the effects of miR-199b-5p on ALK1 expression, cell migration, and tube formation of primary HUVECs *in vitro*, and tumor angiogenesis *in vivo*. We found that miR-199b-5p was downregulated in various breast cancer cell lines and decreased in VEGF-induced HUVECs. Overexpression of miR-199b-5p inhibited the migration and tube formation of HUVECs, while inhibition of miR-199b-5p had the opposite effect. Additionally, overexpression of miR-199b-5p inhibited BMP9-induced activation of the ALK1/Smad/Id1 pathway by binding to the 3’UTR of ALK1. Furthermore, intra-tumoral injection of agomiR-199b-5p suppressed tumor growth and angiogenesis *in vivo.* Taken together, our data suggest that miR-199b-5p may be a potential anti-angiogenic target for cancer therapy.

## Materials and Methods

### Cell Culture

Human umbilical vein endothelial cells (HUVECs) were purchased from Lonza Bioscience (Basel, Switzerland) and cultured in endothelial growth medium-2 (EGM-2) (Lonza). Endothelial basal medium-2 (EBM-2; Lonza) was used for experiments that do not require growth factors. MDA-MB-231 cells obtained from ATCC (VA, USA) were cultured in L-15 medium (Gibco, Carlsbad, USA) supplemented with 10% FBS (Corning, NY, USA), 100 μg/ml streptomycin, and 100 U/ml penicillin. T47D, MCF-7, and BT474 cells obtained from ATCC were cultured in RPMI-1640 medium (Gibco, Carlsbad, USA) supplemented with 10% FBS (Corning, NY, USA), 100 μg/ml streptomycin, and 100 U/ml penicillin. HBL-100 and HEK293T cells obtained from ATCC were cultured in DMEM (Gibco, Carlsbad, USA) supplemented with 10% FBS (Corning, NY, USA), 100 μg/ml streptomycin, and 100 U/ml penicillin. Recombinant human VEGF_165_ (Peprotech, NJ, USA) and recombinant human BMP9 (Peprotech) were added to the expansion medium when needed. The cells were incubated at 37°C in a 5% CO_2_ incubator.

### Cell Transfection

The cells were transfected with agomiR-199b-5p, antagomiR-199b-5p, siALK1, the plasmids pGL3, pRL-TK, and pCMV3-ALK1, respectively, using Lipofectamine 2000 (Invitrogen, CA, USA) according to the manufacturer’s instructions. AgomiR-199b-5p, antagomiR-199b-5p, and siALK1 were synthesized by Shanghai GenePharma Co., Ltd (Shanghai, China). pCMV3-ALK1 (pCMV3-ACVRL1) was purchased from Sino Biological Inc. (Beijing, China).

### Real-Time RT-PCR

Total RNA was isolated from HUVECs using the miRcute miRNA Isolation Kit (Tiangen, Beijing, China), and cDNA was synthesized using the FastQuant RT Kit (Tiangen, Beijing, China) for mRNA analysis. The miRcute microRNA first-strand cDNA synthesis kit (Tiangen, Beijing, China) was used for miRNA analysis. Real-time PCR for mRNA analysis was performed using SuperReal PreMix (SYBR Green) (Tiangen, Beijing, China) and miRNA analysis was performed using miRcute miRNA qPCR Detection Kit (Tiangen, Beijing, China). Following primers were used: ALK1-forward primer, 5’-GACTCAAGAGCCGCAATGTG-3’; ALK1-reverse primer, 5’-GGTCGGCGATGCAACAC-3’; Id1-forward primer, 5’-CTACGACATGAACGGCTGTTA-3’; Id1-reverse primer, 5’-CAACTGAAGGTCCCTGATGTAG-3’; GAPDH-forward primer, 5’-GGAGCGAGATCCCTCCAAAAT-3’; GAPDH-reverse primer, 5’-GGCTGTTGTCATACTTCTCATGG-3’. Primers for has-miR-199b-5p (CD201-0274), has-miR-7 (CD201-0141), has-miR-96 (CD201-0042), has-miR-145 (CD201-0012), has-miR-181a-5p (CD201-0236), has-miR-181b-5p (CD201-0237), has-miR-181c-5p (CD201-0238), has-miR-181d-5p (CD201-0239), has-miR-324-5p (CD201-0346), has-miR-339-5p (CD201-0360), has-miR-874-3p (CD201-0508), has-miR-4262 (CD201-0590), and has-U6 (CD201-0145) were purchased from Tiangen (Beijing, China). GAPDH and U6 were used as controls for miRNA and mRNA detection, respectively.

### Western Blot Analysis

Whole-cell lysates for western blot analysis were extracted with PIPA Lysis Buffer (Beyotime, Jiangsu, China). Antibodies against ALK1 and ID1 were purchased from Abgent Biotechnology (Wuxi, China). Antibodies against p-Smad1/5/8 and Smad1 were obtained from CST Inc. (Danvers, MA, USA). Secondary antibodies conjugated with horseradish peroxidase (HRP) (Sigma-Aldrich, MO, USA) were used for blotting. The blots were visualized using the ECL chemiluminescence reagents from Pierce Biotechnology (Rockford, IL, USA). The quantitation of protein level was analyzed by Image J software.

### Dual-Luciferase Assay

The 3’UTR fragment of ALK1 was cloned into the pGL3 luciferase reporter vector and site mutation of 3’UTR (C to A mutation of target sites) was introduced by GENEWIZ (Beijing, China). Aliquots comprising 100 ng of pGL3-ALK1-WT-3’-UTR, pGL3-ALK1-MUT-3’-UTR, or the empty pGL3 plasmid in combination with agomiR-199b-5p or miRNA scramble negative control, along with 10 ng of pRL-TK plasmid coding *Renila* luciferase were used to co-transfect HEK293T cells. After 36 h post-transfection, luciferase activities were measured using the Dual-Luciferase^®^ Reporter Assay System (Promega Biosciences, LLC., San Luis Obispo, CA, USA) according to the manufacturer’s protocol.

### Tube Formation Assay

HUVECs were transfected with agomiR-199b-5p, antagomiR-199b-5p, miRNA scramble negative control, or agomiR-199b-5p with pCMV3-ALK1 for 48 h and then seeded onto Matrigel (BD, USA)-coated 6-well plates. After 10 h of incubation, network formation was imaged using an inverted optical microscope (Leica, Wetzlar, Germany). The total tube lengths and numbers of intersections in randomly chosen areas were quantified using ImageJ software.

### Transwell Migration Assay

The transwell assay was performed in 6-well transwell plates with polycarbonate membranes (8 μm pore size) (Corning Costar, MA, USA). HUVECs (5 × 10^4^) were transfected with agomiR-199b-5p, antagomiR-199b-5p or miRNA scramble negative control for 24 h and added to the upper compartment of the transwell system in EBM-2 medium. EGM-2 medium was added into the lower chamber. After 24 h, HUVECs that had not migrated on the upper surfaces were cleaned with a cotton swab. Then, the cells on the polycarbonate membranes were fixed and stained with crystal violet (Beyotime, Jiangsu, China) and counted under an inverted optical microscope (Leica, Wetzlar, Germany).

### Wound Healing Assay

HUVECs were seeded into a 6-well plate and transfected with agomiR-199b-5p, antagomiR-199b-5p and miRNA scramble negative control, respectively. After 48 h post-transfection, cells with 90% confluence were pretreated with EGM-2 medium without FBS for 4 h, and then an injury line was made using a sterile 200 μl pipette and unattached cells were washed with PBS twice. Cells were allowed to migrate into the empty space for 24 h in FBS-free EGM-2. The cells were imaged during migration using an inverted optical microscope (Leica, Wetzlar, Germany). The width of the injury was measured using ImageJ software.

### Animal Studies

Female 6-week-old BALB/c nude mice were subcutaneously injected with MDA-MB-231 cells (10^6^) under the armpit. After the tumor volume reached 100 mm^3^, 100 μl of agomiR-199b-5p or miRNA scramble negative control (5 nmol) was injected into the tumor twice a week. Tumor size was measured using the formula: 1/2 × length (mm) × width^2^ (mm) and the weight of the mice was measured before injection. After three weekly injections, the mice (n = 6/group) were sacrificed and tumors were resected for weighing and histological analysis. All animal experiments were performed in accordance with the Guiding Principles for the Care and Use of Laboratory Animals, and all experimental procedures were approved by the Institutional Experimental Animal Committee of Northwestern Polytechnical University (Xi’an, China).

### Histological Analysis

Tissues were fixed in 4% paraformaldehyde, followed by dehydration and embedding with paraffin. The immunofluorescence staining was performed using an anti-CD31 antibody and DAPI according to standard procedures. The stained sections were scanned using an Aperio AT2 Digital Whole Slide Scanner (Leica, Wetzlar, Germany).

### Statistical Analysis

The data were presented as means ± SD. The analysis was performed using GraphPad Prism software. Student’s *t*-test or ANOVA was used to assess the statistical significance. Differences were considered statistically significant at ^*^ *P* < 0.05 or **P < 0.01.

## Results

### MiR-199b-5p, a Possible Regulator of ALK1, was Downregulated in HUVECs During Angiogenesis

**Figure 1:**
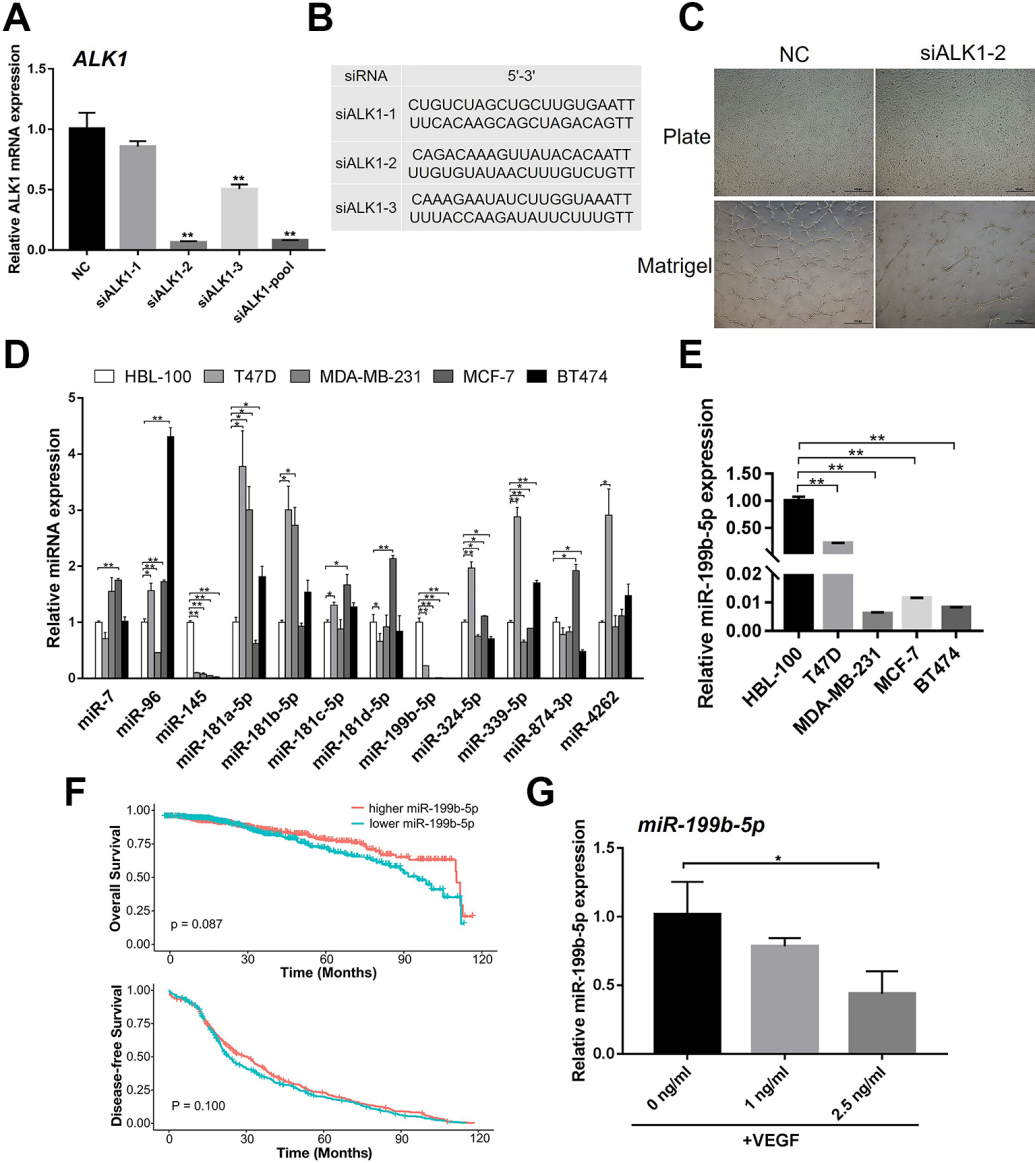
MiR-199b-5p was downregulated during the angiogenesis process of HUVECs. **(A)** Real-time PCR analysis of the knockdown efficiency of ALK1 using siALK1-1, siALK1-2, siALK1-3 and their combination (siALK1-pool) (N = 3). **(B)** The sequences of siALK1-1, siALK1-2, and siALK1-3. **(C)** HUVECs transfected with siALK1-2 were subjected to the tube formation assay in matrigel and culture plate, respectively (N = 3). Representative images of tube formation are presented. Scale bars = 500 μm. **(D)** Real-time PCR analysis of candidate miRNAs possibly targeting ALK1 including miR-7, miR-96, miR-145, miR-181a-5p, miR-181b-5p, miR-181c-5p, miR-181d-5p, miR-199b-5p, miR-324-5p, miR-399-5p, miR-874-3p and miR-4262 in breast cancer cell lines (T47D, MDA-MB-231, MCF-7, and BT474) and a normal breast cell line (HBL-100) (N = 3). **(E)** Enlarged figure of Real-time PCR analysis of miR-199b-5p in breast cancer cell lines (T47D, MDA-MB-231, MCF-7, and BT474) and a normal breast cell line (HBL-100) (N = 3). **(F)** The Kaplan–Meier plot of overall survival and disease-free survival in TCGA database was analyzed according to miR-199b-5p expression. **(G)** Real-time PCR analysis of the expression of miR-199b-5p in HUVECs induced with VEGF (0, 1, and 2.5 ng/ml) for 24 h (N = 3). The data are presented as the means ± SD; *P < 0.05 and **P < 0.01 versus the control group. HUVECs, human umbilical vein endothelial cells; ALK1, Activin receptor-like kinase 1.

It has been reported that ALK1 can positively regulate angiogenesis. Thus, we confirmed the role of ALK1 in HUVECs during angiogenesis. Three small-interfering RNAs (siALK1-1, siALK1-2, and siALK1-3) were designed to specifically target ALK1. HUVECs were transfected with siALK1-1, siALK1-2, siALK1-3, their combination (siALK1-pool), and a scramble siRNA as the negative control. The knockdown efficiency was confirmed by real-time PCR as compared with control cells. The results showed that the mRNA expression of ALK1 was significantly decreased, especially in siALK1-2 transfected cells (knockdown efficiency ~90%) (**Figures 1A, B**). Therefore, siALK1-2 was used to knockdown the expression of ALK1 in HUVECs. At 48 h post-infection, HUVECs were seeded onto matrigel to assess the formation of capillary-like tubes. The result of the tube formation assay showed that knockdown of ALK1 reduced the number of mature and well-connected capillary-like structures (**Figure 1C**).

To identify miRNAs that can potentially target ALK1 to regulate tumor angiogenesis, the bioinformatic tools TargetScan and miRanda were used. We found that miR-7, miR-96, miR-145, miR-181a-5p, miR-181b-5p, miR-181c-5p, miR-181d-5p, miR-199b-5p, miR-324-5p, miR-399-5p, miR-874-3p, and miR-4262 could potentially target ALK1. To further select the ALK1-targeting miRNA that could regulate tumor angiogenesis, we investigated the expression of the identified miRNAs in breast cancer cell lines (T47D, MDA-MB-231, MCF-7, and BT474) and a normal breast cell line (HBL-100). Interestingly, the real-time PCR result showed that the expression of miR-199b-5p was significantly lower in the tested breast cancer cell lines than in the normal breast cell line (**Figures 1D, E**). Based on TCGA database, 10 years survival rate of breast cancer patients (n = 1046) were analyzed in the higher miR-199b-5p group vs the lower miR-199b-5p group. Descriptive statistics of the cases are summarized in **Supplementary Table 1**. Raw data of clinical information of breast cancer patients are in **Supplementary Table 2** The results showed that lower expression of miR-199b-5p is correlated with a poorer overall survival of breast cancer (P = 0.087), while there was no difference in disease-free survival between two groups (**Figure 1F**). In addition, the expression of miR-199b-5p was downregulated in HUVECs during VEGF-induced angiogenesis (**Figure 1G**). These results demonstrated that miR-199b-5p might act as a negative regulator of angiogenesis by targeting ALK1.

### MiR-199b-5p Represses the Capillary Tube Formation of HUVECs

**Figure 2:**
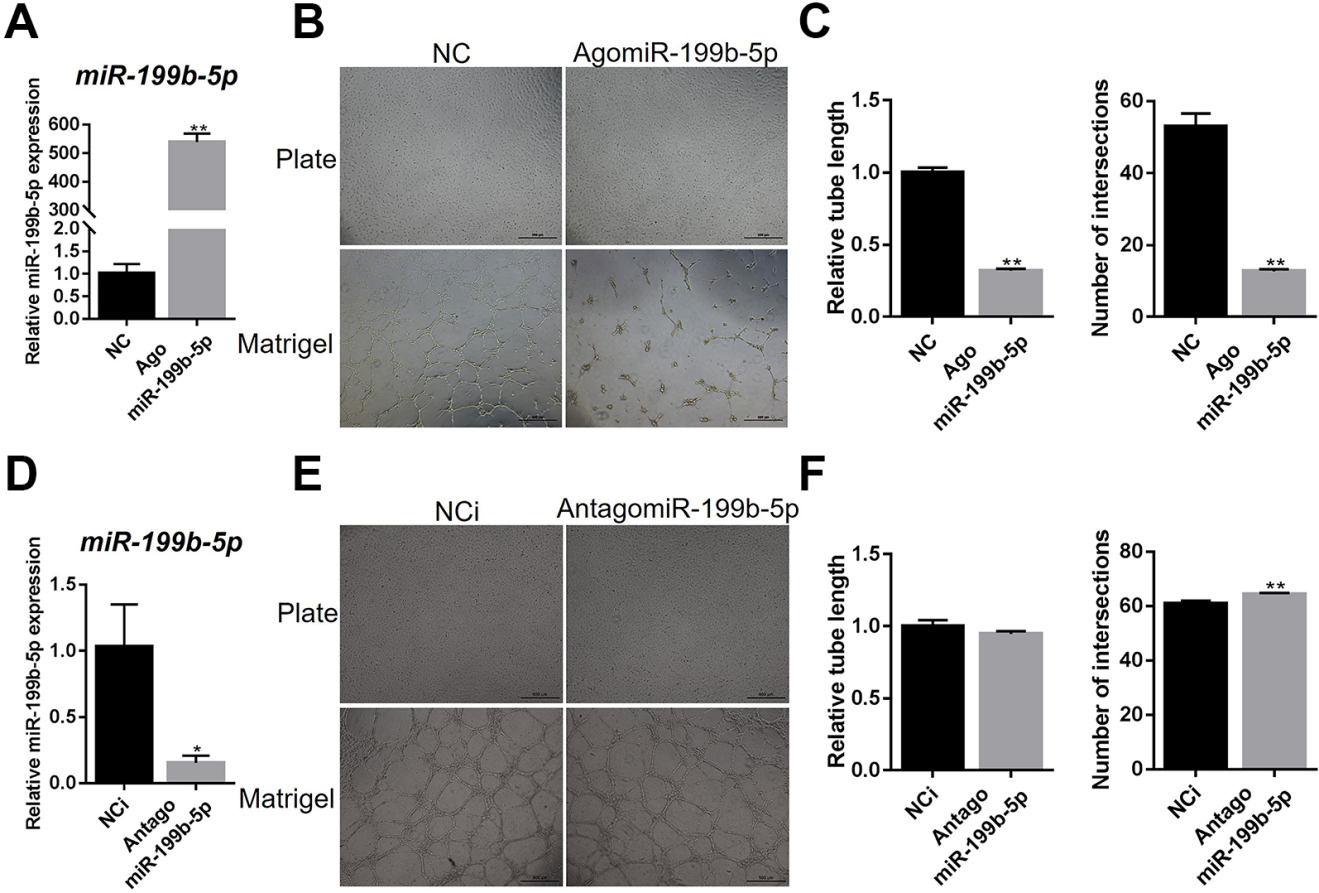
MiR-199b-5p suppresses the tube formation of HUVECs. **(A)** Real-time PCR analysis of miR-199b-5p expression in HUVECs transfected with scramble control and agomiR-199b-5p (N = 3). **(B)** HUVECs transfected with agomiR-199b-5p were subjected to the tube formation assay on matrigel and culture plate, respectively (N = 3). Representative images of tube formation are presented. Scale bars = 500 μm. **(C)** Quantification of tube length and number of intersections in **(B)** using Image J. **(D)** Real-time PCR analysis of miR-199b-5p expression in HUVECs transfected with scramble control and antagomiR-199b-5p (N = 3). **(E)** HUVECs transfected with antagomiR-199b-5p were subjected to the tube formation assay on matrigel and culture plate, respectively (N = 3). Representative images of tube formation are presented. Scale bars = 500 μm. **(F)** Quantification of tube length and number of intersections in **(E)** using Image J. Three independent experiments were performed in triplicate, and the data are presented as the means ± SD; *P < 0.05 and **P < 0.01 versus the control group. HUVECs, human umbilical vein endothelial cells.

To further validate the effects of miR-199b-5p on the angiogenesis on matrigel, we ectopically expressed and inhibited miR-199b-5p in HUVECs by transfecting with agomiR-199b-5p for miR-199b-5p overexpression, antagomiR-199b-5p for miR-199b-5p inhibition, and scramble miRNA as the negative control (**Figures 2A, D**). At 48 h post-transfection, HUVECs were seeded onto matrigel and allowed to form capillary-like tubes. The results showed that overexpression of miR-199b-5p inhibited the angiogenesis process of HUVECs with reduced tube length and a lower number of intersections (**Figures 2B, C**). Conversely, blocking miR-199b-5p increased the number of intersections of the capillary-like tubes (**Figures 2E, F**). Taken together, these experiments demonstrate that miR-199b-5p can modulate the angiogenesis process of HUVECs *in vitro*.

### MiR-199b-5p Represses the Migration of HUVECs

**Figure 3:**
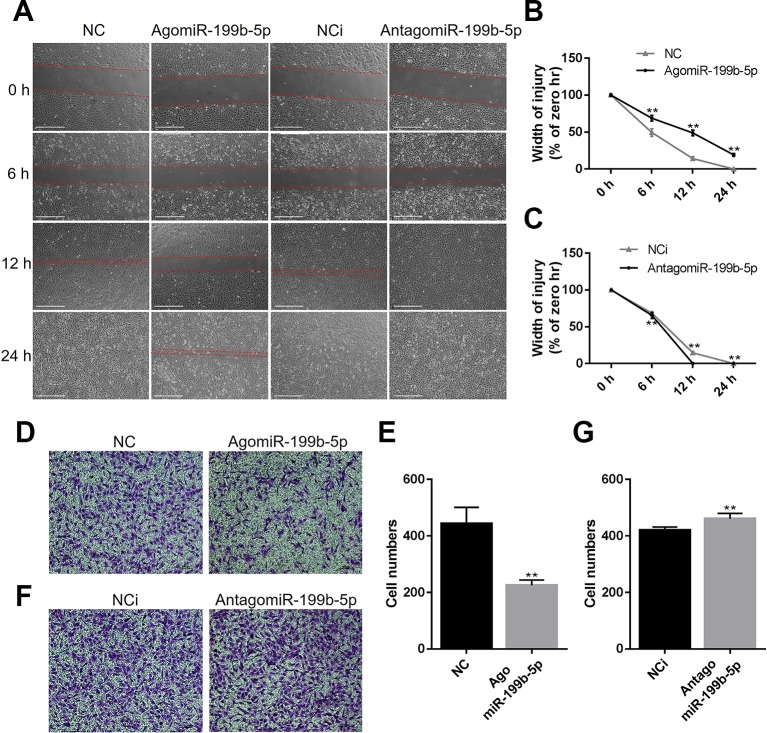
MiR-199b-5p represses the migration of HUVECs. **(A)** Wound healing assay of HUVECs was performed 48 h after transfection with agomiR-199b-5p, antagomiR-199b-5p, and scramble control, respectively. The distance between the wound edges was observed and photographed at 0, 6, 12, and 24 h, respectively (N = 3). Scale bars = 500 μm. **(B**, **C)** The change of the width of injury was evaluated using Image J. **(D**, **E)** Representative and quantified results of the transwell migration assay in HUVECs transfected with agomiR-199b-5p and scramble control. Scale bars = 100 μm. **(F**, **G)** Representative and quantified results of the transwell migration assay in HUVECs transfected with antagomiR-199b-5p and scramble control (N = 3). Scale bars = 100 μm. Three independent experiments were performed in triplicate, and the data are presented as the means ± SD; **P < 0.01 versus the control group. HUVECs, human umbilical vein endothelial cells.

As the migration of endothelial cells is critical for tumor progression, we investigated the effects of miR-199b-5p on the migration of HUVECs using a wound healing assay. The results showed that the scratch healed after 24 h with the migration of HUVECs in the control group, while overexpression of miR-199b-5p reduced the migration rate of HUVECs (**Figures 3A, B**). Conversely, silencing of miR-199b-5p promoted the migration of HUVECs (**Figures 3A, C**).

To further confirm whether miR-199b-5p is involved in regulating the migration of HUVECs, the transwell migration assay was performed. The HUVECs were transfected with agomiR-199b-5p, antagomiR-199b-5p, and scramble miRNA control, and seeded into the upper compartment. After 24 h, the migrated HUVECs were counted following crystal violet staining. The results showed that overexpression of miR-199b-5p significantly suppressed the migration of HUVECs (**Figures 3D**, **E**), while silencing of miR-199b-5p increased cell migration (**Figures 3F, G**). Taken together, these data demonstrated that miR-199b-5p is an important regulator of cell migration in HUVECs.

### MiR-199-5p Directly Targets ALK1 and Thereby Regulates Downstream Genes

**Figure 4:**
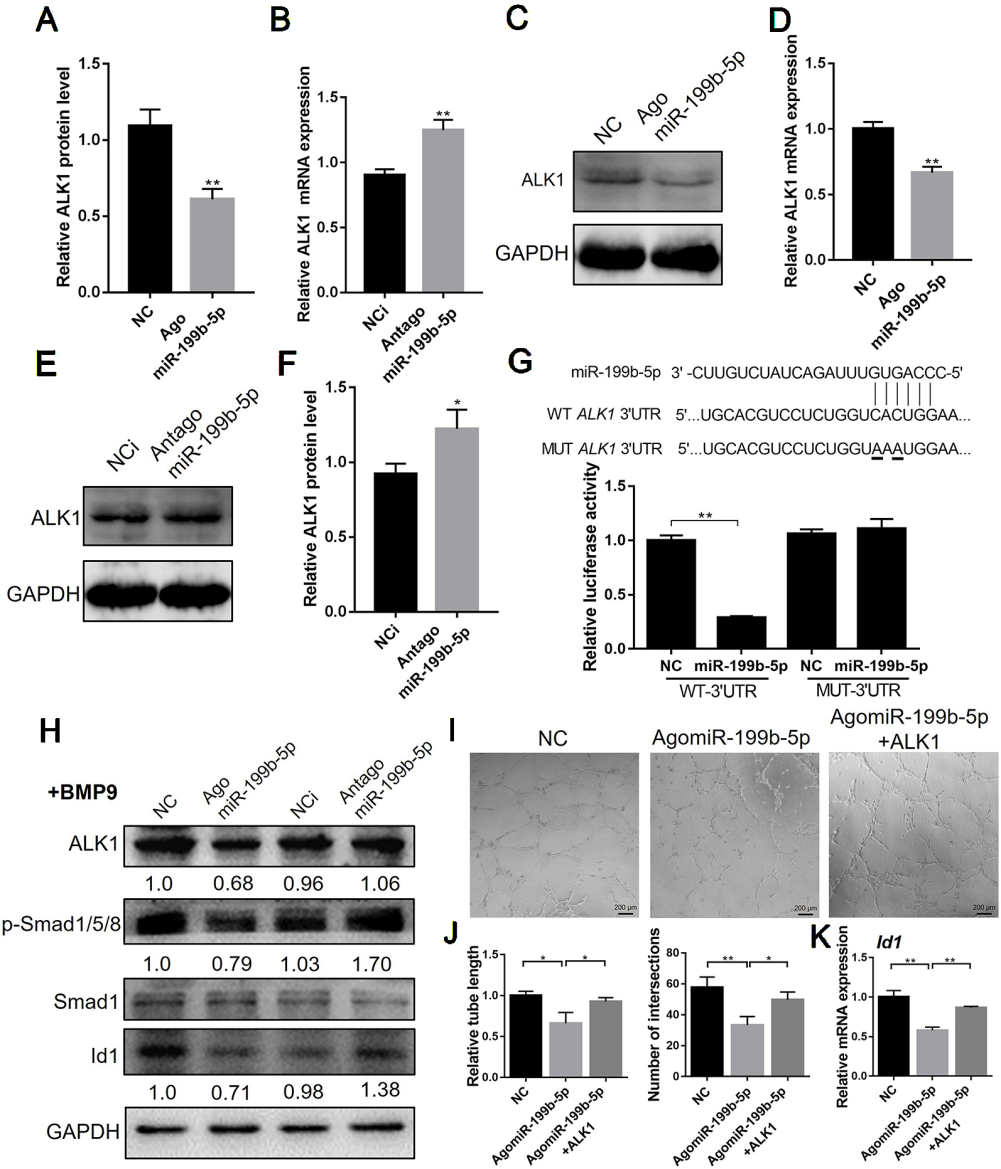
MiR-199-5p directly targets ALK1 and regulate downstream genes. **(A**, **C)** Real-time PCR **(A)** and western blot **(C)** analysis of ALK1 expression in HUVECs transfected with scramble control and agomiR-199b-5p (N = 3). **(B**, **E)** Real-time PCR **(B)** and western blot **(E)** analysis of ALK1 expression in HUVECs transfected with scramble control and antagomiR-199b-5p (N = 3). **(D)** Quantification of relative protein expression in **(C)** using Image J. **(F)** Quantification of relative protein expression in **(E)** using Image J. **(G)** Effects of agomiR-199b-5p on the ALK1 3’UTR luciferase reporters in HEK293T cells. Luciferase activity was measured at 36 hours post transfection (N = 6). **(H)** Western blot analysis of the expression of ALK1, p-Smad1/5/8, Smad1, and Id1 in BMP9-induced HUVECs transfected with agomiR-199b-5p, antagomiR-199b-5p, and scramble control (N = 3). **(I)** HUVECs transfected with agomiR-199b-5p, agomiR-199b-5p with pCMV3-ALK1, and scramble control, were subjected to the tube formation assay on matrigel, respectively (N = 3). Representative images of tube formation are presented. Scale bars = 200 μm. **(J)** Quantification of tube length and number of intersections in **(I)** using Image J. **(K)** Real-time PCR analysis of Id1 expression in HUVECs transfected with agomiR-199b-5p, agomiR-199b-5p with pCMV3-ALK1, and scramble control on matrigel (N = 3). The data are presented as the means ± SD; *P < 0.05 and **P < 0.01 versus the control group. HUVECs, human umbilical vein endothelial cells; ALK1, Activin receptor-like kinase 1.

Bioinformatic analysis using TargetScan and miRanda predicted that miR-199b-5p targets the 3’UTR of ALK1. Thus, we firstly examined the effect of miR-199b-5p on the mRNA and protein expression of ALK1. The real-time PCR results showed that overexpression of miR-199b-5p decreased the mRNA expression of ALK1 (**Figure 4A**). Conversely, silencing miR-199b-5p increased ALK1 expression in HUVECs (**Figure 4B**). The results of western blot analysis confirmed that overexpression of miR-199b-5p inhibited the protein expression of ALK1 (**Figures 4C, D**), while knocking down miR-199b-5p had the opposite effect (**Figures 4E, F**). Taken together, these data indicate that miR-199b-5p can regulate ALK1 expression at the post-transcriptional level.

To confirm that miR-199b-5p directly targets the 3’UTR of ALK1, we introduced full-length sequences of the wild-type or mutated human ALK1 3’UTR into the pGL3 luciferase reporter vector. The binding of miR-199b-5p to the 3’UTR of ALK1 was detected using a dual-luciferase reporter assay in HEK293 cells. The results showed that overexpression of miR-199b-5p significantly repressed WT ALK1 3’UTR luciferase activity, while the mutation of the miR-199b-5p binding site abolished the inhibition of luciferase activity (**Figure 4G**). These results suggest that miR-199b-5p directly targets the 3’UTR of ALK1. To investigate the effect of miR-199b-5p on downstream signaling of ALK1, miR-199b-5p overexpressing and knockdown HUVECs were serum-starved for 4 h and stimulated with 10 ng/ml bone morphogenetic protein 9 (BMP9) for 30 min. The results of western blot analysis showed that overexpression of miR-199b-5p could inhibit the BMP9-induced phosphorylation of Smad1/5/8 and Id1 expression in HUVECs (**Figure 4H**). Furthermore, we investigated whether overexpression of ALK1 could rescue the inhibition of miR-199b-5p on angiogenesis of HUVECs. HUVECs were transfected with agomiR-199b-5p, agomiR-199b-5p with pCMV3-ALK1 plasmid, and scramble miRNA as the negative control. At 48 h post-transfection, HUVECs were seeded onto matrigel and allowed to form capillary-like tubes. The analysis of relative tube length and number of intersections showed that the reduced angiogenic activity of miR-199b-5p overexpressed HUVECs was restored by ALK1 overexpression (**Figures 4I, J**). The real-time PCR results showed that downregulation of Id1 in miR-199b-5p overexpression HUVECs was rescued by ALK1 overexpression during angiogenesis (**Figure 4K**). These results indicated that miR-199b-5p could attenuate angiogenesis by directly targeting ALK1 in HUVECs.

### MiR-199-5p Reduces Tumor Growth and Angiogenesis *In Vivo*

**Figure 5:**
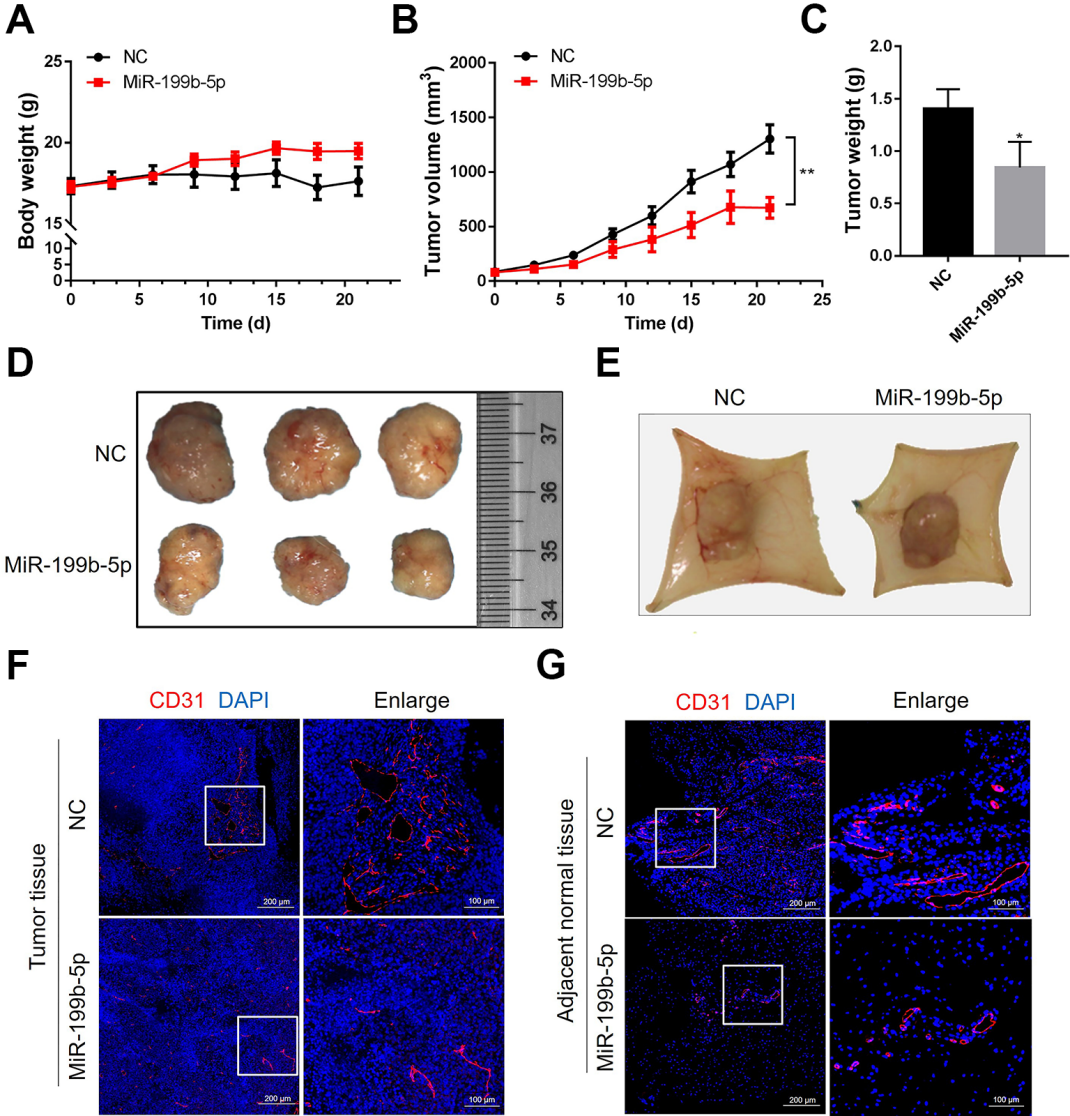
MiR-199-5p reduces tumor growth and angiogenesis *in vivo*. **(A)** The body weight of mice from the scramble control treatment and agomiR-199b-5p treatment was measured every three days (N = 6). **(B)** A comparison of tumor size between the agomiR-199b-5p group and the scramble control group (N = 6). **(C)** Tumor weights of mice from the scramble control treatment and agomiR-199b-5p treatment groups were measured after three weekly injections (N = 6). **(D)** Representative images of tumors isolated from mice treated with scramble control and agomiR-199b-5p by surgical excision on the final day of the experiment. **(E)** Representative images of capillary tubes within tissues adjacent to the tumor on the final day of the experiment. **(F**, **G)** Representative images of immunofluorescence detection of blood vessels (red, CD31 staining) in tumors **(F)** and adjacent tissues **(G)** using an Aperio AT2 Digital Whole Slide Scanner. The data are presented as the means ± SD; *P < 0.05 and **P < 0.01 versus the control group.

Since the *in vitro* experiments showed that miR-199b-5p inhibits the angiogenic activity and migration of HUVECs, we further investigated whether miR-199b-5p could inhibit tumor angiogenesis and growth *in vivo*. MDA-MB-231 cells were subcutaneously injected into NOD/SCID mice. After the tumors reached a volume of 100 mm^3^, agomiR-199b-5p, and scramble miRNA control were injected intratumorally twice a week for 3 weeks. The body weight and tumor growth were measured every 3 days. The body weight of the mice in the miR-199b-5p treatment group slowly increased compared to control group (**Figure 5A**), suggesting a therapeutic effect of miR-199b-5p with low toxicity to tissues and organs. Additionally, the growth of the subcutaneous tumors injected with agomiR-199b-5p was significantly suppressed (**Figure 5B**).

Following 21 days of treatment, the tumors and adjacent tissues were harvested and photographed. The weight of the tumors injected with agomiR-199b-5p was significantly lower than that of tumors injected with scramble miRNA control (**Figure 5C**). Consistently, the average size of the tumors from the agomiR-199b-5p group was smaller than that of those from the scramble miRNA control group (**Figure 5D**). Furthermore, the effect of miR-199b-5p on angiogenesis was also analyzed. The image of tumor tissue showed that miR-199b-5p treatment reduced the formation of neovasculature within tumor tissues and capillary tubes within adjacent tissues (**Figure 5E**). The density of CD31-positive vessels in tumor sections was analyzed using immunofluorescence staining with an antibody against CD31. The results showed that miR-199b-5p reduced the number and size of blood vessels in tumors and adjacent tissues compared to scramble miRNA control (**Figures 5F, G**). Taken together, these results demonstrate that miR-199b-5p could suppress tumor growth and tumor angiogenesis *in vivo*.

## Discussion

**Figure 6:**
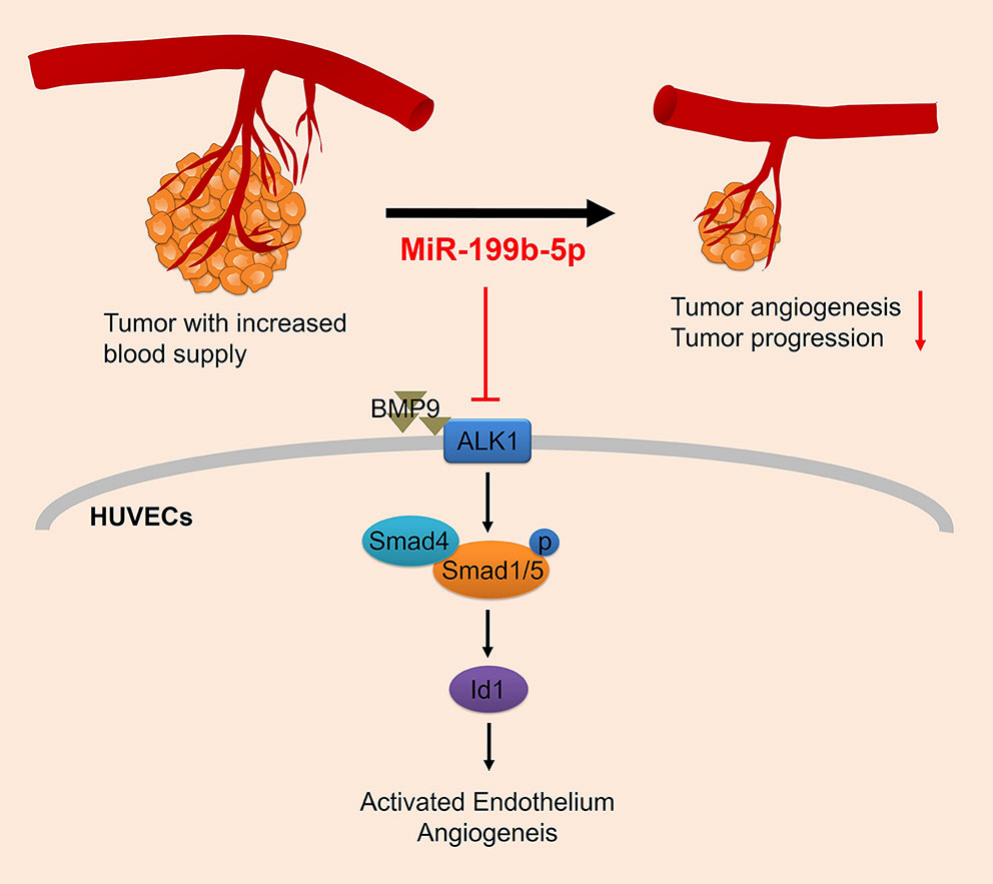
Proposed regulatory model depicting the mechanism of miR-199b-5p in suppressing tumor angiogenesis by targeting ALK1. HUVECs, human umbilical vein endothelial cells; ALK1, Activin receptor-like kinase 1.

Angiogenesis is critical to tumor growth and metastasis. In this study, we identified for the first time that miR-199b-5p is a negative regulator of angiogenesis in HUVECs. Overexpression of miR-199b-5p significantly inhibited the migration and tube formation of HUVECs *in vitro* and tumor angiogenesis *in vivo*. It was also found that miR-199b-5p could directly target ALK1 to negatively regulate ALK1/Smad/Id1 signaling in HUVECs. Therefore, we conclude that miR-199b-5p could suppress tumor angiogenesis by targeting ALK1 and the ALK1/Smad/Id1 signaling pathway may be involved in the mechanism (**Figure 6**).

Tumor angiogenesis is critical for the progression from initial tumor growth to metastasis. Since Folkman reported that angiogenesis is required for the growth of solid tumors in 1971 (Folkman, 1971), anti-angiogenic strategies were developed as an effective cancer therapies. The first antiangiogenic agent bevacizumab, an anti-VEGF antibody, was found to be effective in patients with metastatic colorectal cancer (Ferrara et al., 2004; Ronca et al., 2017). Beyond that, many anti-angiogenic agents have been developed based on angiogenesis inducers, including VEGF, bFGF, IL-8, and PDGF (Rajabi and Mousa, 2017). ALK1 is predominantly expressed in endothelial cells (Roelen et al., 1997). Homozygous ALK1^−/−^ mice died at embryonic days 10–10.5 due to vascular and cardiac abnormalities (Oh et al., 2000). Genetic studies revealed that ALK1 plays a key role in vasculogenesis and organization of neo-angiogenic vessels (Hu-Lowe et al., 2011). Additionally, the expression of ALK1 is upregulated in large arteries during tumor angiogenesis (Mitchell et al., 2010). BMP9/10-ALK1 signaling has recently also been identified as a target for the development of anti-angiogenic therapies (Jonker, 2014; de Vinuesa et al., 2016). Immunological sequestration of ALK1 ligands (using ALK1-Fc) or antibody against ALK1 have been reported to inhibit tumor angiogenesis and tumor growth in mouse models of pancreatic cancer, breast cancer, and melanoma (Roman and Hinck, 2017). Therefore, ALK1 emerged as a potential therapeutic target for anti-angiogenic therapy of diverse solid tumors.

MiRNAs are increasingly being investigated as potential anti-angiogenic small molecule drugs for tumor therapy (Goradel et al., 2019). In this study, bioinformatic analysis was used to identify miRNAs that could potentially target ALK1, and 12 candidate miRNAs were selected. Among these, miR-199b-5p and miR-145-5p were found to be significantly downregulated in four different breast cancer cell lines compared to normal breast cells. MiR-145-5p was found to inhibit the growth of pancreatic cancer and angiogenesis *in vivo* by suppressing angiopoietin-2 (Wang et al., 2016), a critical regulator of tumor angiogenesis and progression (He et al., 2014). It has been demonstrated that miR-145 is a tumor suppressor in angiogenesis and tumor growth (Xu et al., 2012; Zou et al., 2012; Arderiu et al., 2019).

However, the effect of miR-199b-5p on tumor angiogenesis is unclear. It has been reported that low miR-199b-5p levels are associated with a poor prognosis in breast cancer (Fang et al., 2016), distant metastasis in colorectal cancer (Shen et al., 2016), and invasion of lymph nodes or perineural invasion in head and neck squamous cell carcinoma (Sousa et al., 2016). Our results showed that miR-199b-5p expression is reduced in breast cancer cells, which was in agreement with these earlier findings. MiR-199b-5p was found to reduce the aggressiveness of papillary thyroid carcinoma cells (Ren et al., 2019) and inhibit the migration and invasion of head and neck squamous cell carcinoma cells (Koshizuka et al., 2017). Therefore, miR-199b-5p might be involved in carcinogenesis. Tumor cells and endothelial cells are neighboring cells in tumor microenvironment that take part in angiogenesis and cancer metastasis. Extracellular vesicles, such as exosomes, can deliver miRNAs from tumor cells to endothelial cells to modulate recipient cells (Zhang et al., 2015). Reports showed that exosomal miRNAs, such as miR-25-3p and miR-221-3p, from tumor cells can promote angiogenesis in HUVECs (Zeng et al., 2018; Wu et al., 2019). It has been demonstrated that miR-199b-5p can influence the angiogenesis of mouse myocardial microvascular endothelial cells (Du et al., 2016). However, the function of miR-199b-5p in the angiogenesis process of HUVECs and tumor angiogenesis, as well as its target genes, have not yet been reported. In our study, we found that overexpression of miR-199b-5p could inhibit the angiogenesis process of HUVECs on matrigel with a reduction of tube length and number of intersections. Conversely, knockdown of miR-199b-5p increased the intersection number of the capillary tubes in HUVECs. Similarly, knockdown of ALK1 in HUVECs using siRNAs, resulted in decreased angiogenesis. Additionally, the wound healing assay and transwell migration assay showed that overexpression of miR-199b-5p significantly suppressed the migration of HUVECs, and silencing of miR-199b-5p had the opposite effect. These findings support the role of miR-199b-5p in the migration and angiogenesis process of HUVECs.

Mechanistically, we found that elevated miR-199b-5p expression was accompanied by significantly decreased mRNA and protein expression of ALK1. To identify the target sequence of miR-199b-5p in the 3’UTR of ALK1, a dual-luciferase reporter assay was used. The results showed that overexpression of miR-199b-5p significantly repressed the luciferase activity of WT ALK1 3’UTR, while a mutation of the miR-199b-5p binding site abolished the inhibition of luciferase activity. During the angiogenesis process of HUVECs, BMP9 can induce downstream genes including Smad1/5/8 and Id1 through ALK1 (van Meeteren et al., 2012). Western blot analysis revealed that ALK1, phospho-Smad1/5/8, and Id1 were downregulated after transfection with miR-199b-5p under BMP9 induction in HUVECs, as expected. Moreover, overexpression of ALK1 could rescue the inhibition of miR-199b-5p on angiogenesis of HUVECs. These data revealed that miR-199b-5p could directly target ALK1 and mediate the downstream ALK1/Smad1/5/8/Id1 signaling pathway during angiogenesis of HUVECs.

Finally, we investigated the effect of miR-199b-5p on tumor angiogenesis *in vivo*. The size and weight of the tumors were reduced in the group that was intratumorally injected with agomiR-199b-5p. In agreement with the *in vitro* results that overexpression of miR-199b-5p inhibits tube formation of HUVECs, the miR-199b-5p treatment caused a reduction of the number and size of blood vessels in tumors and adjacent tissues.

In conclusion, our present study highlights that miR-199b-5p is a newly identified inhibitor, of the migration and angiogenesis process of HUVECs *in vitro*, as well as tumor angiogenesis and tumor progression *in vivo* through the silencing of ALK1 expression. Our results suggest that upregulating or delivering miR-199b-5p might be a potential strategy for the treatment of tumors by suppressing angiogenesis.

## Data Availability Statement

The data that support the findings of this study are available from the corresponding author upon reasonable request.

## Ethics Statement

The animal study was reviewed and approved by the Institutional Experimental Animal Committee of Northwestern Polytechnical University (Xi’an, China).

## Author Contributions

XL and AQ designed the experiments. XL, WQ, YX, and AQ performed experiments. XL, YG and YL collected data. XL, WQ, YX, JM, and KZ analyzed data. XL and AQ wrote the manuscript. XL, YL, FX, HL, QC, and AQ revised the manuscript. All authors approved the final version of the manuscript.

## Conflict of Interest

The authors declare that the research was conducted in the absence of any commercial or financial relationships that could be construed as a potential conflict of interest.
