# Supplementary material for: MiR-199b-5p Suppresses Tumor Angiogenesis Mediated by Vascular Endothelial Cells in Breast Cancer by Targeting ALK1
Source: Front Genet. 2020 Jan 30;10:1397. doi: 10.3389/fgene.2019.01397 (PMC7002562; doi:10.3389/fgene.2019.01397)
Supplement: Supplementary file 1 [file Table_1.docx]

**Supplementary table 1. Clinicopathological characteristics of the patients cohort (n = 1046)**

| **Characteristics** |  | **n** | **%** |
| --- | --- | --- | --- |
| **Age (years)** | 26~29 | 10 | 0.95 |
|  | 30~39 | 66 | 6.31 |
|  | 40~49 | 211 | 20.17 |
|  | 50~59 | 276 | 26.39 |
|  | 60~69 | 268 | 25.62 |
|  | 70~79 | 148 | 14.15 |
|  | 80~90 | 67 | 6.41 |
| **Menopause status** | Pre | 225 | 21.51 |
|  | Post | 675 | 64.53 |
|  | Peri | 39 | 3.73 |
| **Metastasis pathologic pm** | NA | 107 | 10.23 |
|  | M0 | 860 | 82.22 |
|  | M1 | 20 | 1.91 |
|  | MX | 160 | 15.30 |
|  | Cm0(i+) | 6 | 0.57 |
| **Nodes pathologic** | N0 | 484 | 46.27 |
|  | N1 | 349 | 33.37 |
|  | N2 | 118 | 11.28 |
|  | N3 | 77 | 7.36 |
|  | NX | 18 | 1.72 |
| **Stage** | Stage I | 170 | 16.25 |
|  | Stage II | 595 | 56.88 |
|  | Stage III | 241 | 23.04 |
|  | Stage IV | 19 | 1.82 |
|  | Stage X | 11 | 1.05 |
|  | NA | 10 | 0.96 |
| **Tumor pathologic** | T1 | 269 | 25.72 |
|  | T2 | 614 | 58.70 |
|  | T3 | 127 | 12.14 |
|  | T4 | 35 | 3.35 |
|  | TX | 1 | 0.09 |
| **Cancer type** | Breast Invasive Ductal Carcinoma | 770 | 73.61 |
|  | Breast Invasive Lobular Carcinoma | 198 | 18.93 |
|  | Breast Invasive Mixed Mucinous Carcinoma | 15 | 1.43 |
|  | Breast Mixed Ductal and Lobular Carcinoma | 27 | 2.58 |
|  | Invasive Breast Carcinoma | 6 | 0.57 |
|  | Metaplastic Breast cancer | 14 | 1.34 |
|  | Other types | 16 | 1.53 |
| **ER status** | Positive | 232 | 22.18 |
|  | Negative | 768 | 73.42 |
|  | NA | 46 | 4.40 |

*NA represents samples without related data.
